# Overview and emerging trends

**DOI:** 10.4103/0019-5545.43052

**Published:** 2005

**Authors:** Sandeep Grover, Ajit Avasthi, Subho Chakrabarti, Paramanand Kulhara

**Affiliations:** * Senior Resident, Department of Psychiatry, PGIMER, Chandigarh; ** Professor, Department of Psychiatry, PGIMER, Chandigarh; *** Associate Professor, Department of Psychiatry, PGIMER, Chandigarh; **** Professor, Department of Psychiatry, PGIMER, Chandigarh

## INTRODUCTION

The economic impact of mental disorders is wide-ranging, long-lasting and huge. These disorders impose a range of costs on individuals, families and communities as a whole.^1^ Economic evaluation is concerned with the best use of limited resources and occurs in a decision-specific context of identifying the most efficient way of meeting a stated objective. Its main function is to allow policy-makers, managers and clinicians to make choices by assessing the costs and benefits of achieving objectives by different methods. Health care budgets are limited. However, there is no limit to expenditure if all existing demands are to be met. Finite resources and the discrepancy between demand and the available supply suggest that a formula for allocating resources among the various competing sectors is necessary. A choice has to be made between differing treatments, treatment settings and illnesses to allow the judicious use of scarce resources.^2^ Therefore, measuring the economic burden imposed by mental illnesses on the family and society has been an important endeavour. Several studies have tried to document the cost in terms of treatment expenses, family spending, loss of manpower, etc.

Schizophrenia is a burdensome illness, with implications not only for patients and families, but also for the health services and other care agencies.^3^ While the majority of patients recover from their psychotic symptoms following their first episode of illness, they are subsequently at a higher risk for relapse, and ultimately for persistent morbidity in the form of residual positive or negative symptoms or both, neurocognitive impairment and deficits in social, occupational and vocational functioning.^4^ In addition, patients with schizophrenia also die prematurely compared with the general population, due to increased risk for suicide, poor living conditions, poor nutrition or poor access to health care services.^5^ Several studies have assessed the family burden of schizophrenia in India, and have reported that financial burden is common to them all.^6^–^9^ This was more so when the affected person was an earning member of the household.^6^ The obvious reasons for this increased financial burden can be early age of onset, which may lead to lifelong disability; disease chronicity, which may result in long-term morbidity; hospitalization and maintenance drug therapy; and social and economic effects on caregivers, such as expenditure incurred due to extra arrangements, loans taken or savings spent and putting off any planned activity because of the financial pressure of the patient's illness.^10^ Financial constraints lead to poor drug compliance and relapse, which further perpetuates the financial burden.

## METHODS OF ECONOMIC EVALUATION

There are several methods of economic evaluation. These include cost-minimization analysis, cost-effectiveness analysis, cost–benefit analysis, cost-utility analysis, and cost of illness (COI) analysis. Among the various methods, COI studies describe the economic burden of disease on society. COI studies also allow for comparison between different illnesses. From the health system perspective, only direct medical costs are relevant. From a societal perspective, all costs are relevant including direct medical costs, direct non-medical costs and indirect costs. COI studies emphasize the issue of cost containment as well as the benefits of total elimination of the disease. COI studies can draw attention to disorders with an overall high burden; disorders with poor investment in services and other resources used in their treatment; the possible impact of preventive measures on primary, secondary and tertiary prevention; comparison with other disorders in a common currency. COI studies are also are important in estimating the economic burden in that costs of health service intervention reflect the existing pattern of service delivery.^2^

A COI study can be based on either prevalence or incidence rates, and employs either a top–down or bottom–up approach. The underlying rationale of the prevalence-based method is that costs are assigned to the year in which they are borne, or with which they are directly associated. This type of costing identifies the major contributors to the current expenditure. Expected future earnings lost as a result of premature death are assigned to the year of death. If cost control is the aim of the exercise, this approach allows for the identification of possible targets for economizing. A prevalence-based COI study gives an idea of the magnitude of the problem, and can be useful for priority-setting on a macro level. If a change in service delivery is being implemented, the prevalence approach allows comparison of the balance sheets before and after the change. It can also be of great help in comparing the societal burden of two diseases. However, there is no link to benefit from a possible intervention to reduce the burden of the disease. The incidence approach is based on the principle that the flow of costs associated with the disease should be assigned to the year in which that flow begins. All future direct and indirect costs are estimated and discounted so that they can be measured in monetary terms of the year in which the illness first occurs, because once the illness has occurred the society at one level or another is committed to meet the streams of cost that will be associated with that illness. This approach is useful because it can predict the likely long-term impact of programmes that reduce incidence, make treatment less expensive or improve outcome. An incidence-based COI study is helpful when one has to decide between alternative methods of interventions, because an intervention will affect all future treatment costs and productivity losses.^11^

While estimating the COI, most studies use either of two computational methods to determine the direct costs of disease: a ‘top–down’ or a ‘bottom–up’ approach. In the top–down approach, the entire health care expenditure is calculated and the share attributable to a particular illness is then determined.^12^ The bottom–up approach uses a defined subpopulation of the disorder for costing, and the costs are then extrapolated to give total population costs. The various approaches which have been used to assess indirect costs include a human capital-base approach, willingness-to-pay or contingent valuation-base approach and a friction cost--base approach. The human capital approach views individuals as producing a stream of output that is valued at market rates, and the value of life is the discounted future earnings. The willingness-to-pay approach values life according to what individuals are willing to pay for a change that reduces the probability of illness or death. This is more difficult to measure since it takes into account the perception of pain and suffering associated with a condition. Friction costs represent the costs associated with the replacement of a sick worker. The concept behind the use of friction costs is that production losses due to illness may not be as great as expected, because existing labour pools and workplace structures can absorb some of this lost productivity. Friction costs include costs associated with the amount of time needed to replace a sick worker, training costs for new or temporary employees, and costs associated with any decrease in productivity during temporary work absence of the sick employee, or from the substitution of the workforce needed to replace the sick employee.^2^,^12^

## COMPONENTS OF COST PACKAGES

Parts of this economic burden are obvious and measurable, while other parts are almost impossible to measure. Among the measurable components of the economic burden are health and social service needs, lost employment and reduced productivity, impact on families and caregivers, levels of crime and public safety, and the negative impact of premature mortality. The parts that cannot be measured in monetary terms are called intangible costs and includes effects on the patients in the form of stigma, stress and side-effects of treatment, and on the caregivers in the form of stress, psychiatric morbidity and stigma.^1^ All relevant costs are usually examined to assess the economic impact of a mental health care intervention. The various components of cost estimation are direct costs, indirect costs, hidden costs and non-measured or intangible costs.

*Direct costs* are the actual monetary expenditure related to treating an illness or disorder, i.e. these include costs associated with hospitalization, outpatient services, nursing care, drugs, services of a range of professionals, residential care, day care, and domiciliary care and rehabilitation.^2^,^13^ They include provider's cost which is the cost borne by the hospital for providing medical facilities.^2^

*Indirect costs* concern the monetary value of lost output due to reduced or lost productivity of patients and caregivers, caused by illness, disability or injury of patients,^14^ family costs in looking after a sick relative, and cost of various allowances.^2^ Some authors also include costs associated with public awareness campaigns, crime control and health insurance, and losses due to premature death.^15^

*Intangible costs* cannot be expressed in monetary terms, and include effects on the patient in the form of stigma, stress, and side-effects of treatment; and on the caregiver in terms of stress, stigma and psychiatric morbidity.^2^

## RESEARCH ON THE COST OF ILLNESS IN SCHIZOPHRENIA

A number of studies have attempted to calculate the cost of care of schizophrenia in developed countries. The findings of these studies vary widely because of methodological dissimilarities. In contrast, there are only a few studies of COI from developing countries in which comprehensive costing has been undertaken.^2^ Findings from developed countries have shown that the COI of schizophrenia varies from 1.6% to 2.5% of the annual health care budgets.^11^,^16^–^19^ In monetary terms, the cost has varied from US$ 2.35 billion to US$ 3270 billion per year for all patients of schizophrenia depending on the type of methodology and year of study.^3^,^16^,^17^,^20^–^22^ Many studies have compared direct and indirect costs and the results have shown wide variation in the percentage attributed to each, depending on the type of study. Direct costs have ranged from 13% to 53% of the total cost, and indirect costs from 47% to 87%.^3^,^11^,^16^,^18^–^34^

On the whole, however, different authors have claimed that either the proportion of direct costs and indirect costs are nearly equal,^35^,^36^ or that indirect costs are three to four times higher.^16^,^21^,^23^ Studies have constantly shown that drug costs form a small fraction of the total cost, varying from 2% to 5.6% of the total cost;^28^,^37^ and from 3% to 5% of the direct cost.^38^ The COI of schizophrenia has been compared with both physical and psychiatric illnesses, and the consistent finding is that the cost of care of schizophrenia is much more than that of other illnesses.^23^,^35^,^39^ Studies from developed countries have also shown that various factors influence the COI of schizophrenia, but the findings are inconclusive. Some authors have found no positive association between any of the demographic parameters and costs of treatment.^14^,^40^ Others have reported higher costs among men^25^,^33^,^35^ or women,^19^ in the young^17^,^25^,^33^,^41^–^44^ as well as the old.^45^ Living alone, being single or unemployed have all been linked to increased total, direct or indirect costs,^46^ but on the other hand there are studies which have found higher costs for patients who live with others and are unemployed.^33^ Among the clinical variables, some reports have suggested that a longer duration of illness leads to higher costs.^33^,^46^ Moscarelli *et al*.^47^ found that the length of time between the onset of illness and first contact/admission was a significant determinant of total costs. Studies have also shown higher costs for patients with a higher number of inpatient episodes in the past.^33^,^41^–^44^ However, the most consistent associations with the costs of care across several studies are of severity of illness and levels of disability. Treatment costs are significantly higher among severely ill patients, or those with impaired functioning.^18^,^22^,^33^,^40^,^48^,^49^

### Cost of illness studies from developing countries

Unfortunately, information from developing countries is scarce although the illness is as common and perhaps as disabling. The few studies that are available suggest that there could be differences in the types of costs incurred, which is expected, given the vast differences in sociocultural and treatment milieus. Suleiman *et al.*^14^ estimated the monetary cost of treating a group of Nigerian outpatients with schizophrenia in comparison with insulin-dependent diabetes mellitus and found that the cost of schizophrenia was significantly less than that of diabetes mellitus. This was largely due to the cost of insulin injections, needles and syringes. The cost of antipsychotic drugs accounted for 52.8% of the total cost of schizophrenia, while insulin injections accounted for 92.8% of the total cost of diabetes mellitus. Patients with schizophrenia and their relatives suffered significantly more loss of working days.

### Cost of illness studies from India

There are very few studies which have evaluated the cost of mental illnesses in India. Girish *et al.*^50^ found that antipsychotic drugs are affordable and are comparable to drug treatment costs of other physical illnesses. They found that the monthly cost of treatment with chlorpromazine was Rs *55,* an equivalent dose of trifluperazine amounted to Rs *25* per month, risperidone Rs *60* and clozapine Rs *225* per month. They also found that there was a marked price difference across brands. They concluded that although antipsychotic drugs are affordable, the other costs associated with treatment make them more expensive; these could be co-prescribed antiparkinsonian agents, antidepressants, anxiolytics, etc. Sarma showed that cost of one outpatient visit was Rs *201* in which the contribution of the management was 68% and the patient's contribution 32%;^51^ it was found that salaries accounted for a maximum proportion, i.e. 48% of the total cost, this was followed by loss of earnings, which accounted for 17%. Drugs accounted for less than 10% of the total cost. Chisholm *et al.,* screened four rural populations in India and Pakistan for psychiatric morbidity.^52^ Individuals with a diagnosable mental disorder were invited to seek treatment and assessed prospectively on symptoms, disability, quality of life and resource use. Seventy-two per cent of cases in Bangalore and 92% of cases in Rawalpindi belonged to the broad category of mood disorders. They found that at baseline, the cost of treatment in the Bangalore site was Rs *700* per month and in the Rawalpindi site it was more than Rs *3000* per month. The total cost was equivalent to between *7* and 14 days of an agricultural worker's wages in India, and approximately 20 days' wages in Pakistan. These total costs decreased appreciably by the follow-up assessment point in 3 of the 4 localities.

We conducted a study on COI of schizophrenia with the hope that such evaluation would increase awareness of the costs incurred by patients, families and treating agencies caring for patients with schizophrenia.^53^ Cost estimation was loosely based on the prevalence-based, bottom–up approach. Indirect costs were calculated on the basis of the human capital approach principle. Though costs were primarily assessed based on a survey of patients, data were also obtained from the hospital records and statistics. To increase the reliability and comprehensiveness of cost estimates, information obtained from patients was supplemented by information from caregivers. As most patients in India are likely to receive treatment as outpatients in general hospital clinics, only outpatient treatment costs were calculated. The results of the study showed marked differences when compared with the data from developed and other developing countries. The total annual cost of care of schizophrenia amounted to Rs 13,687.38, which was not significantly different from that of diabetes mellitus. The total indirect costs of schizophrenia were Rs 8620.44 annually. Loss of earnings of patients (46.50% of the total costs) and caregivers (11.89% of the total costs) accounted for almost all of this, the remainder being made up of other expenditures such as expenses on the patient's recreation, interest on loans, etc. (4.58% of the total costs). The total direct costs of schizophrenia (excluding provider's costs) amounted to Rs 4460.88 annually. Drug costs and travel costs accounted for the bulk of such costs. The hospital provided a few medicines to patients with schizophrenia; the cost of these was negligible (0.68%) compared with what was spent by patients. Money spent by patients on buying drugs constituted about 18% of the total costs. Expenses incurred on travel by patients and their families comprised 8.56% of the total costs. The cost of alternative treatments amounted to 3.9% of the total costs. Another important finding of our study was that the expenses incurred by patients worked out to be slightly less than half (48.31%) of their total income per month. The cost of services provided by the hospital in this study formed only a negligible proportion (4.42%) of the total costs.

Employment status was the only sociodemographic parameter that had a significant influence on the costs of care. Total, as well as direct, costs were significantly higher among the unemployed in this group. The number of visits made to the hospital demonstrated significant positive correlations with total, direct, indirect and provider's costs in both groups of patients. Duration of illness did not correlate significantly with costs. The association of costs with clinical severity and levels of disability were by far the most impressive. There was a strong correlation between the Positive and Negative Symptom Scale (PANSS) scores and Schedule for Assessment of Psychiatric Disability (SAPD) scores with total as well as various other components of costs.

This study demonstrates that schizophrenia is an expensive illness in monetary terms, even in developing country settings. Some important conclusions can be drawn from the above study. First, the cost of treatment of schizophrenia in India is different from that reported from many developed countries and hence there is a definite need for such studies to plan health services. Second, the cost of care of schizophrenia is no different from that of other chronic physical illnesses such as diabetes mellitus. There is a great need to generate awareness of this fact among all manner of professionals involved in the care of patients with schizophrenia, as well as health policy-makers. Third, the study also demonstrates that the main brunt of the financial burden caused by schizophrenia is borne by the impoverished family; hence the financial burden on the family is much higher compared with western figures. The recent World health report^1^ showed that in several developing countries with low-income economies including India, the government's share of health expenditure was diminishing, and costs were being increasingly borne by individuals and households. In developing countries, almost all patients stay with their families. Moreover, the lack of adequate community mental health facilities means that such relatives are often left to care for mentally ill persons all by themselves. This further magnifies the already large financial burden that they have to bear. Any efforts at cost reduction, therefore, should have the family as the primary focus. Fourth, patients in our country have to buy all the drugs themselves, and hospitals supply only a few cheap drugs, free of cost and thus the drug costs are generally higher compared with developed countries. Fifth, the more severe the disorder, the higher the cost of management. This implies that the disorder should be managed appropriately at the beginning to avoid the development of severe disability. A similar picture has also been reported by Davies and Drummond^16^ who found that 97% of the total lifetime costs of schizophrenia were incurred by fewer than 50% of the patients with severe chronic illnesses, multiple disabilities, and excessive dependence on services. This is known as the ‘funding–imbalance’ effect. Since disability and severity of illness contribute to higher costs, reductions in symptoms and improvement in functioning will be paralleled by a reduction in costs. Therefore, treatments that improve outcome are more likely to reduce costs and be cost-effective for patients with schizophrenia as well. Sixth, the number of visits made to the hospital is an important determinant of the total COI. Hence, reducing the number of visits to the hospital can be one of the most effective ways of cutting down on costs.

This study has not answered all the issues. First, about three fourths of the patients in this study were urban-based, which meant that they did not have to spend much on travelling to a hospital located in the city. Most of the subjects in the study were local inhabitants and did not have to spend anything on stay near the hospital for treatment. However, since mental health facilities are unevenly distributed in developing countries, patients often have to travel long distances and arrange for accommodation near the treating agency. This inevitably increases the amount spent on such ventures. Second, the costs of inpatient treatment were not covered by the study, which can increase the cost manifold. Third, since indirect costs are often more difficult to compute, they have not been measured in totality. Mortality costs and other costs, e.g. those of caregiving, were not measured in the current study. Therefore, indirect costs were likely to have been underestimated. Moreover, it has been proposed that the human capital approach used in this study does not adequately measure indirect costs; alternative approaches have been suggested.

## CONCLUSION

Changing economic trends have meant that evaluation of treatment costs is no longer a subject of mere academic interest, even in the developing world. The need for such studies is obvious, relevant and practical. This is because pharmacoeconomics is likely to become an important basis for health policy decisions as a number of important dynamics evolve in the marketplace. These include consumers acting on their growing access to information and becoming more actively involved in treatment decisions; payers, providers and patients deepening their interaction and overcoming their traditional focus on either costs or benefits alone; and manufacturers being challenged by other health care constituencies as sponsors of cost-based outcome studies. In the same vein, several authors have pointed out that since costs of schizophrenia are context-bound, these might differ from country to country (even in the developing world), based on differences in economic indices, e.g. per capita income, local demography, as well diversity in cultures, social structures, and health care services. Hence, a cost analysis of the existing health care system can help to identify inefficiencies in the system and the expenditure pattern. The need for information on costs in India assumes greater importance as the Government of India is now considering implementing user fees for health care services. In India, information on costs of health services usually comes from budgetary statements. The actual costs of various components of the health care delivery system are often not available. This is because budgetary provisions cover only recurrent expenditures such as salary and drugs but do not reflect the investments already made in the form of infrastructure and equipment, costs borne by the family and the work hours lost due to absence from duty and disability. Hence, there is a need for more studies on the pharmaco-economics of mental illnesses. It is hoped that in the long run such studies will facilitate improved allocation of the resources available for the treatment of mental illness in India.
